# Targeted gene expression profiling for accurate endometrial receptivity testing

**DOI:** 10.1038/s41598-023-40991-z

**Published:** 2023-08-26

**Authors:** Alvin Meltsov, Merli Saare, Hindrek Teder, Priit Paluoja, Riikka K. Arffman, Terhi Piltonen, Piotr Laudanski, Mirosław Wielgoś, Luca Gianaroli, Mariann Koel, Maire Peters, Andres Salumets, Kaarel Krjutškov, Priit Palta

**Affiliations:** 1 Competence Centre On Health Technologies, 50411 Tartu, Estonia; 2 Department of Genetics and Cell Biology, GROW School for Oncology and Developmental Biology, Maastricht University, 6200 MD Maastricht, The Netherlands; 3 Department of Obstetrics and Gynecology, Institute of Clinical Medicine, University of Tartu, 50406 Tartu, Estonia; 4 Institute of Biomedicine and Translational Medicine, University of Tartu, 50411 Tartu, Estonia; 5 Department of Obstetrics and Gynecology, PEDEGO Research Unit, Medical Research Center, Oulu University Hospital, University of Oulu, FI-90014 Oulu, Finland; 6 Oviklinika Infertility Center, 01-377 Warsaw, Poland; 7 Women’s Health Research Institute, Calisia University, 62-800 Kalisz, Poland; 8 Department of Obstetrics, Gynecology and Gynaecological Oncology, Medical University of Warsaw, 02-091 Warsaw, Poland; 9 Medical Faculty, Lazarski University, Warsaw, Poland; 10 SISMeR, Reproductive Medicine Institute, 40138 Bologna, Italy; 11 Institute of Genomics, University of Tartu, 51010 Tartu, Estonia; 12 Division of Obstetrics and Gynecology, Department of Clinical Science, Intervention and Technology (CLINTEC), Karolinska Institutet and Karolinska University Hospital, SE-141 52 Stockholm, Sweden; 13 Institute for Molecular Medicine Finland (FIMM), University of Helsinki, FI-00014 Helsinki, Finland

**Keywords:** Molecular biology, Biomarkers, Molecular medicine, Reproductive disorders, Gene expression

## Abstract

Expressional profiling of the endometrium enables the personalised timing of the window of implantation (WOI). This study presents and evaluates a novel analytical pipeline based on a TAC-seq (Targeted Allele Counting by sequencing) method for endometrial dating. The expressional profiles were clustered, and differential expression analysis was performed on the model development group, using 63 endometrial biopsies spanning over proliferative (PE, n = 18), early-secretory (ESE, n = 18), mid-secretory (MSE, n = 17) and late-secretory (LSE, n = 10) endometrial phases of the natural cycle. A quantitative predictor model was trained on the development group and validated on sequenced samples from healthy women, consisting of 52 paired samples taken from ESE and MSE phases and five LSE phase samples from 31 individuals. Finally, the developed test was applied to 44 MSE phase samples from a study group of patients diagnosed with recurrent implantation failure (RIF). In validation samples (n = 57), we detected displaced WOI in 1.8% of the samples from fertile women. In the RIF study group, we detected a significantly higher proportion of the samples with shifted WOI than in the validation set of samples from fertile women, 15.9% and 1.8% (p = 0.012), respectively. The developed model was evaluated with an average cross-validation accuracy of 98.8% and an accuracy of 98.2% in the validation group. The developed beREADY screening model enables sensitive and dynamic detection of selected transcriptome biomarkers, providing a quantitative and accurate prediction of endometrial receptivity status.

## Introduction

Infertility affects millions of people of reproductive age worldwide, and increasingly more couples undergo in vitro fertilisation (IVF) to achieve pregnancy^1^. Though IVF success rates have improved significantly, many patients still fail to conceive and experience recurrent implantation failure (RIF). The reasons for embryo implantation failure are highly heterogeneous, attributable to the quality of the embryo, the endometrium, and the interaction between the two^2^. As these circumstances vary from patient to patient, applying a personalised approach for assessing embryo quality and endometrial receptivity could potentially increase the chance of implantation during IVF.

The period when the endometrium becomes receptive to embryo implantation is called the window of implantation (WOI). There is no consensus yet on the exact timing and length of the WOI period. Generally, the endometrium is considered to become receptive seven days after the luteinising hormone (LH) peak during the natural cycle (NC) and lasts for two days. However, several studies suggest that the length of the WOI may vary from two to up to 6 days^3–6^. In addition, based on transcriptional studies of endometrial biopsies, the WOI can be shifted or displaced over time^7,8^. It has been estimated that displaced WOI occurs in around 10% of women undergoing IVF and at least 25% with RIF^6,9–11^. In this light, the personalised determination of the WOI timing could be a standard procedure for women undergoing IVF. This would be crucial to avoid implantation failure after embryo transfer, possibly due to the asynchrony between the endometrial and embryonal development. Therefore, testing the endometrial receptivity and establishing the time for WOI may be particularly beneficial for RIF patients.

Polycystic ovary syndrome (PCOS) is an infertility-associated disorder common among women of reproductive age. It is still unclear whether endometrial receptivity is affected in PCOS patients. The dysregulation of several endometrial receptivity-associated genes in PCOS patients has been previously described^12^. Besides the altered gene expression profile of endometrial tissue, PCOS patients are often affected by obesity, hyperinsulinemia, and increased general inflammation, likely contributing to infertility^13^. Nevertheless, to our knowledge, no systematic studies have been published describing the gene expression signature of essential endometrial receptivity genes in PCOS throughout the entire menstrual cycle.

Considerable effort has been made to describe molecular changes in the WOI^14,15^. The hormone-induced regulatory cascade leads to endometrial maturation and major gene expression changes, culminating with the opening of the WOI. The transcriptomic landscape of endometrial maturation is well characterised through whole transcriptome studies where different gene expression profiles have been detected between proliferative (PE), early- (ESE), mid- (MSE), and late-secretory (LSE) endometrium^14,16,17^. Based on these studies, biomarkers for WOI determination and personalised embryo transfer (pET) have been provided. Diagnostic tests based on gene expression profiling of varying sets of endometrial receptivity biomarkers have been developed and integrated into infertility treatment^18–21^. Endometrial receptivity tests currently in clinical use are the ERA^®^ test (Igenomix)^18^, the ER Map^®^ test (IGLS)^20^, the WIN-Test (INSERM)^21^, and the rsERT test (Yikon Genomics Company)^10^. Compared to the targeted sequencing approach used in the present study, these tests are limited either by the sensitivity and dynamic range in detecting transcript abundances or by the scalability of the technique applied.

**Figure 1:**
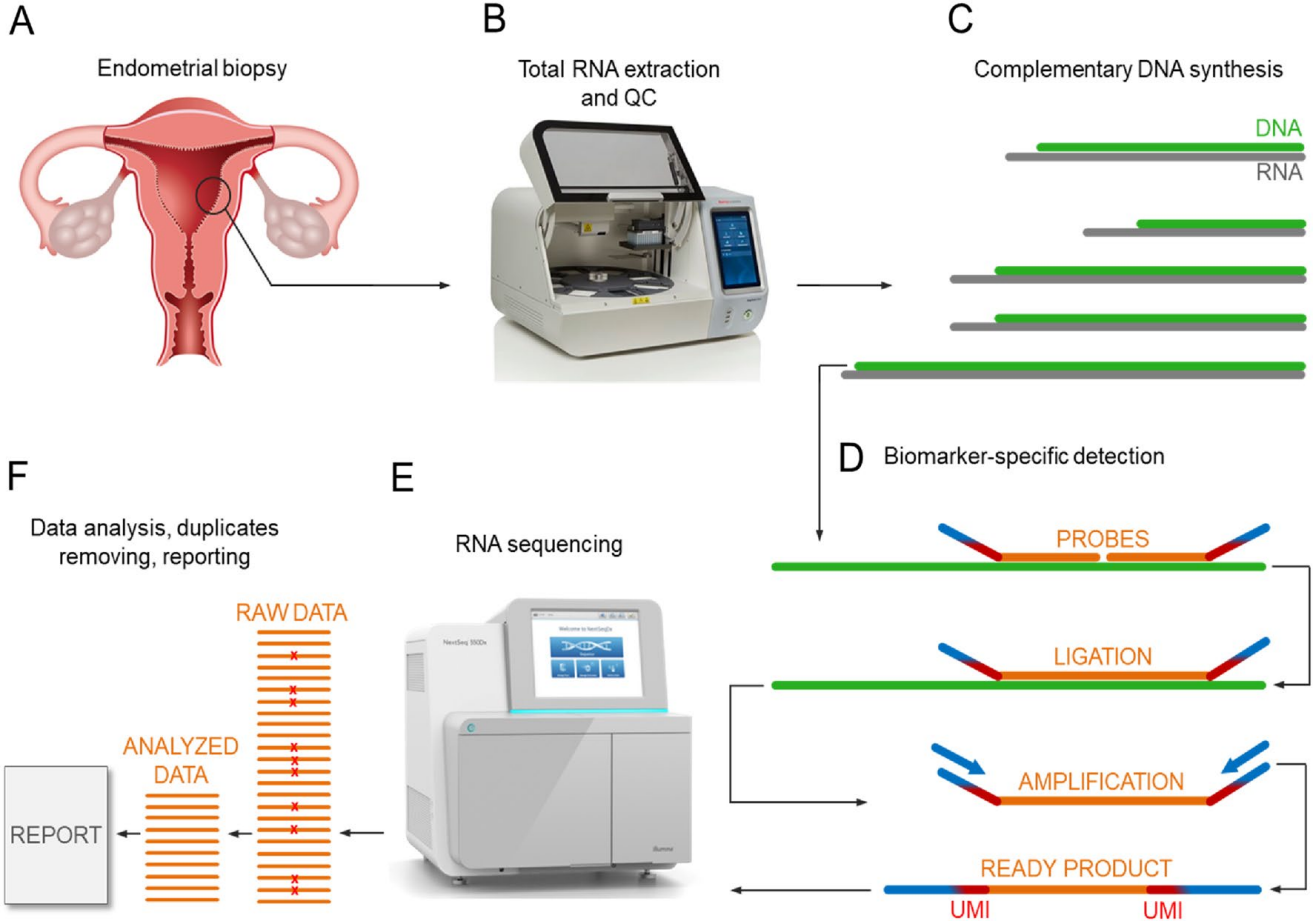
Illustrative schematic overview of beREADY endometrial receptivity biomarker analysis by TAC-seq technology. (**A**) Endometrial biopsy is the source of total RNA that contains a set of analysed biomarker genes. (**B**) The biopsy’s visual quality control, total RNA extraction, and quality control (QC). (**C**) The set of selected biomarkers’ RNA molecules in grey for the template of complementary DNA in green after oligo-T priming. (**D**) Biomarker-specific detection through DNA-DNA hybridisation with orange probes. The ligation joints the probe DNA molecules only in a perfect match. The blue arrows represent PCR primers introducing patient-specific barcodes that copy red UMI parts and orange biomarker regions for RNA sequencing. (**E**) Illumina RNA sequencing technology generates millions of reads per analysed sample. (**F**) The orange RNA sequencing reads represent raw data that includes technical PCR duplicates marked with red crosses. Further bioinformatic analysis recognises and removes the duplicates based on UMI-s and profiles the sample of interest naturally. The final step is a report.

This study introduces the beREADY model for reliable WOI detection. The 72 genes analysed with this test contain 57 endometrial receptivity-associated biomarkers^22^, 11 additional genes relevant to WOI, and four housekeeper genes. The computational model was developed using Illumina sequencing-based TAC-seq technology (Targeted Allele Counting by sequencing), enabling biomolecule analysis down to a single-molecule level^23^, as shown in Fig. 1.

## Results

### Study design

First, gene expression signatures of 68 endometrial receptivity genes were analysed in the model training and development (MD) group samples (n = 63), consisting of both healthy volunteers and PCOS patients. Based on collected data, a continuous and quantitative three-stage (from pre-receptive to receptive to post-receptive) computational classification model was developed. Next, the model classification accuracy was validated on the model validation (MV) group samples (n = 57, including ESE, MSE, and LSE samples) of healthy women. Finally, the RIF group samples (n = 44, women with RIF in the natural cycle (NC) at MSE) were examined with the validated computational classification model. The outline of this study is presented in the Supplementary Fig. 1.

### Gene expression profiling of PCOS samples

**Figure 2:**
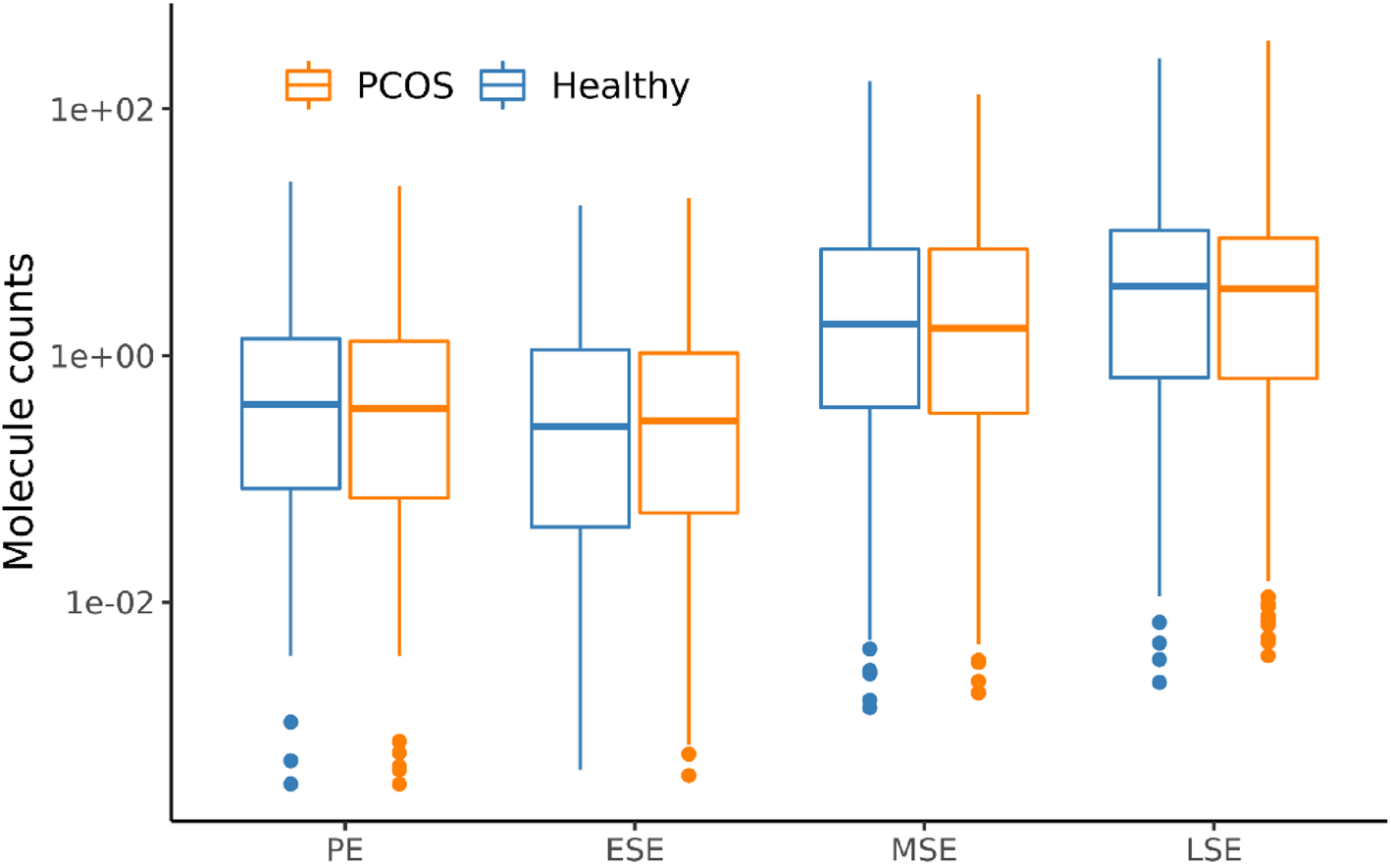
Gene expression profiles of endometrial biomarker genes in samples of polycystic ovarian syndrome (PCOS) patients and healthy women. The average gene expression (per group) of all assayed and housekeeper-normalised genes is presented for healthy and PCOS samples in the MD group. The lower and upper hinges of the boxplot correspond to the 25th and 75th percentiles. *PE* proliferative phase, *ESE* early-secretory phase, *MSE* mid-secretory phase, *LSE* late-secretory phase and *MD* model development.

This analysis of endometrial receptivity biomarkers revealed no difference between healthy women and PCOS patients (Fig. 2). Comparative t-testing and ANOVA of individual genes did not reveal any significantly (FDR < 0.05) differentially expressed genes between the groups of PCOS patients and healthy women (Suppl. Table 1). These results were observed in all four menstrual cycle phases (Fig. 2) and confirmed by principal component analysis (Suppl. Fig. 2). Based on these results, we concluded that PCOS status does not affect the expression profiles of biomarkers included in the developed assay.

### beREADY model development

**Figure 3:**
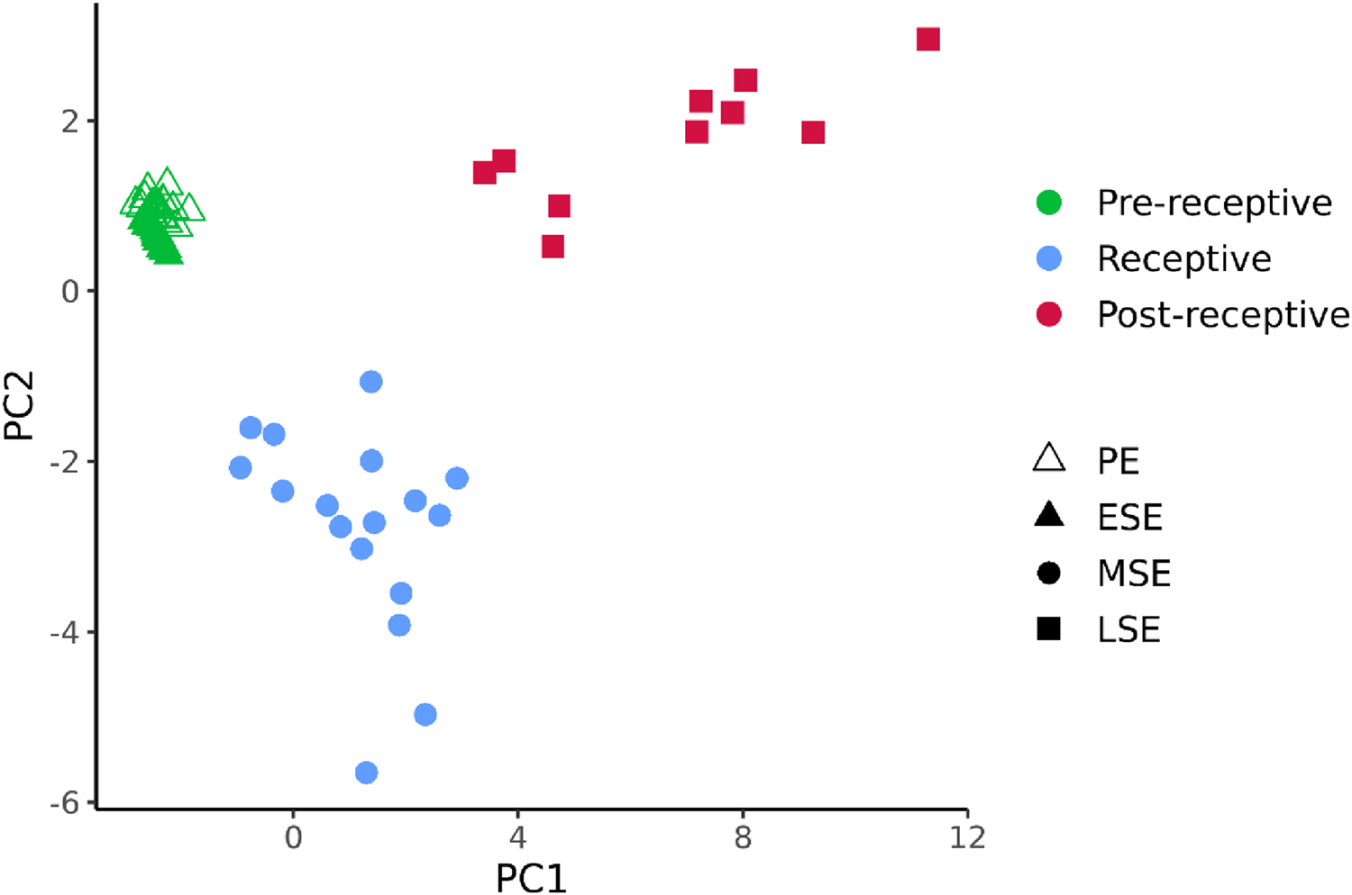
PCA plot of the model development group detects distinct transcriptional profiles of endometrial phases. The UMI-corrected counts were normalised with the geometric mean of housekeepers and scaled. *PE* proliferative phase, *ESE* early-secretory phase, *MSE* mid-secretory phase, *LSE* late-secretory phase, *PCA* principal component analysis and *UMI* unique molecular identifier.

**Figure 4:**
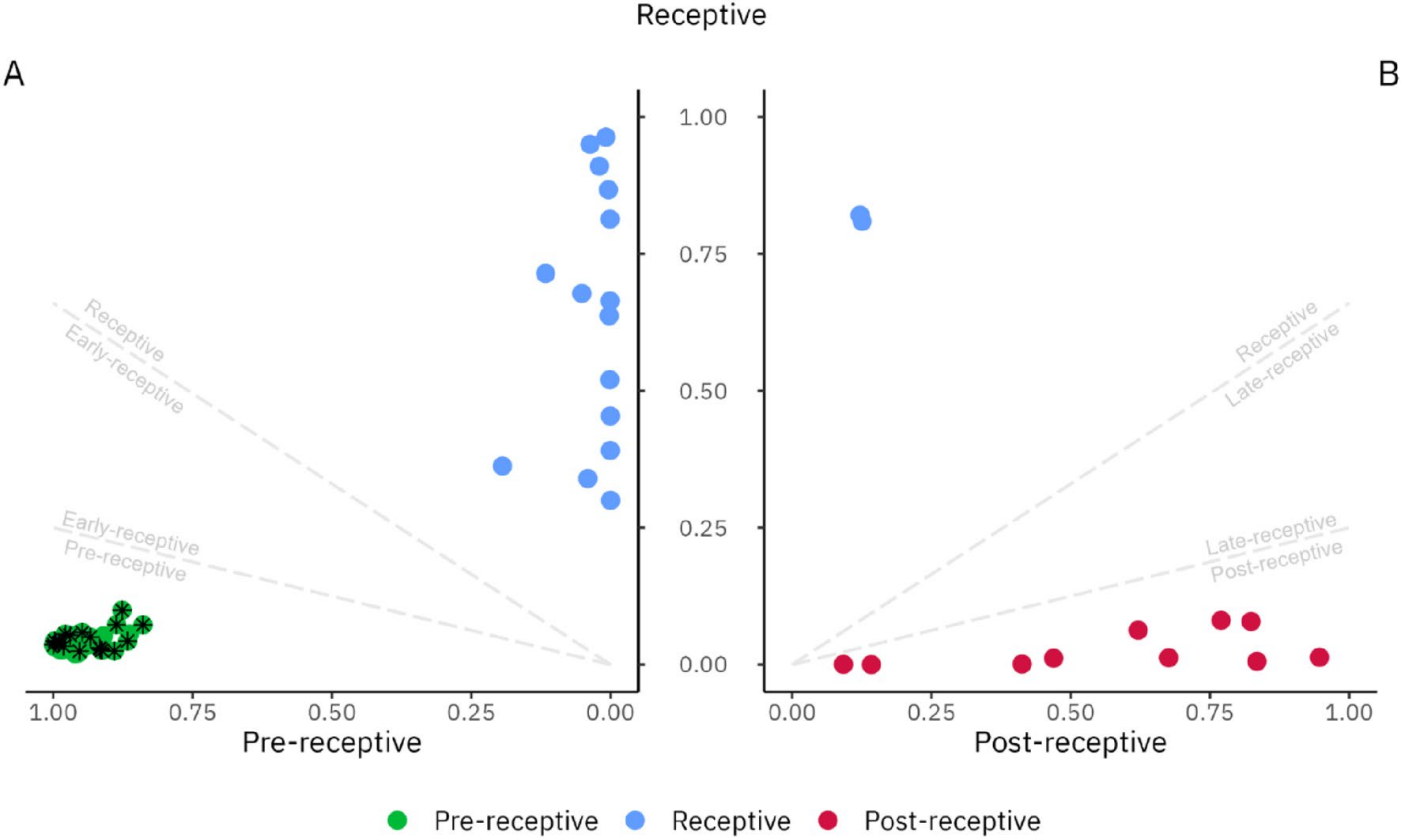
beREADY model output probabilities for model training and development group (MD) samples that belong to pre-receptive, receptive and post-receptive groups. The dashed lines represent intermediary groups as early-receptive and late-receptive decision boundaries, which are not detected in this analysis step. (**A**) The clustering between pre-receptive and receptive groups. Proliferative samples are marked with an asterisk. (**B**) The clustering between receptive and post-receptive groups.

Before test development, 11 samples from the MD group were excluded from downstream analysis due to inconsistency between histology results and LH-day measurements. The remaining 63 MD group samples clustered clearly according to the menstrual cycle phases and enabled determining the receptivity class prediction (Fig. 3). When the PE phase and ESE samples were analysed together, the signature of receptivity genes allowed the discrimination of three groups of pre-receptive (PE and ESE samples), receptive (MSE samples), and post-receptive (LSE) samples (Suppl. Fig. 3). According to predicted menstrual cycle phase probabilities, the MSE endometria (n = 17) were defined as receptive, corresponding to WOI. The PE and ESE endometrial samples (n = 36) were classified as pre-receptive, suggesting that the endometrium has not yet reached the WOI. The LSE endometria (n = 10) were defined as post-receptive, reflecting the period after WOI (Fig. 4). Based on fivefold cross-validation, the predictive model classified PE/ESE, MSE, and LSE samples with 98.8% accuracy, averaged over all the receptivity classes.

Endometrial samples with two similar receptivity classes were classified as belonging to a transitionary class. Samples between the pre-receptive and receptive groups were defined as early-receptive and samples between receptive and post-receptive as late-receptive. However, samples positioned in the early-receptive, receptive, or late-receptive groups were collectively considered to represent the normal variability of WOI.

### beREADY model validation

**Figure 5:**
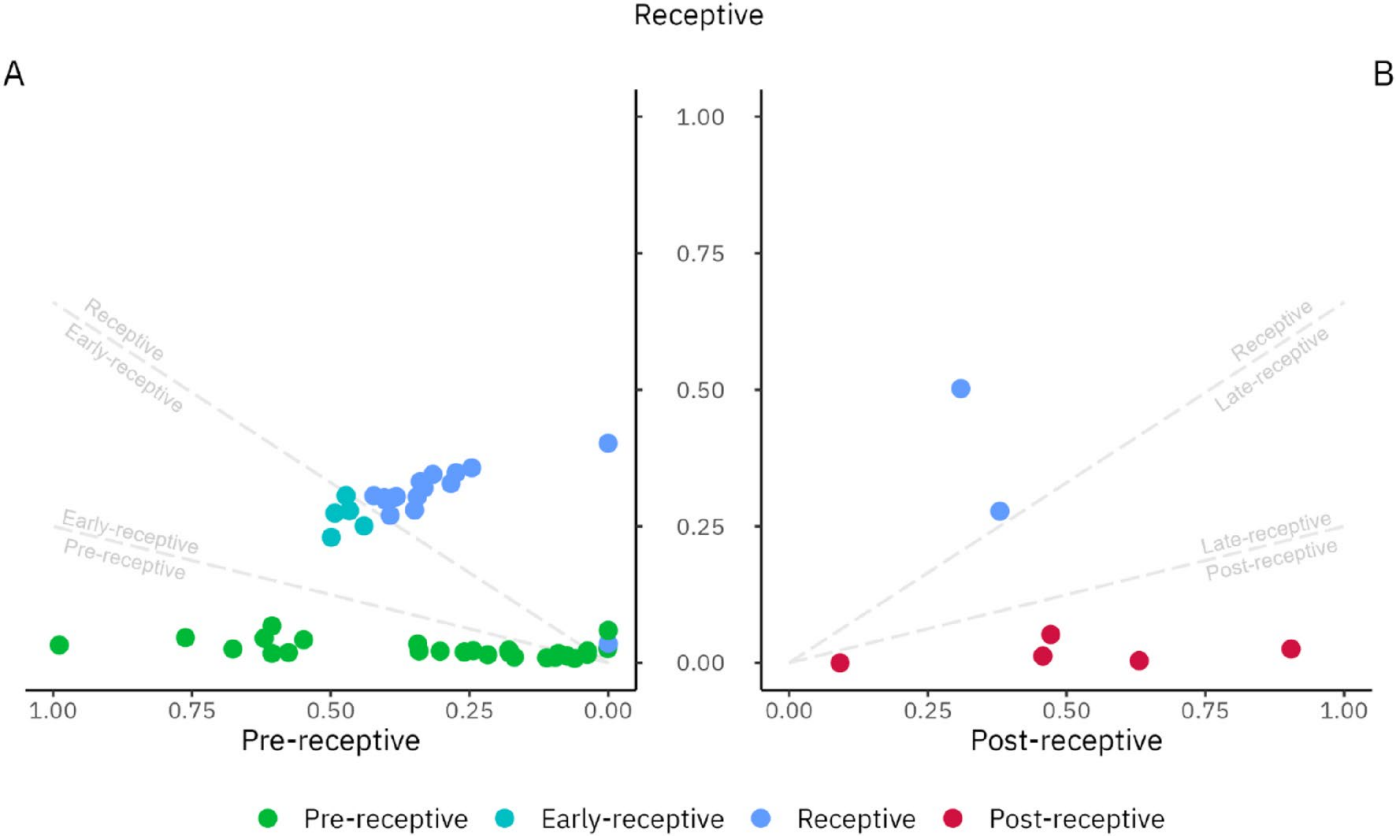
A focused analysis of the model validation (MV) samples, revealing early-receptive samples in mid-secretory group. Each point on this plot represents the probability of belonging to the given class after the first cluster distancing. The colours represent the final prediction class for given samples. (**A**) Comparative pre-receptive and receptive group analysis revealed the intermediary early-receptive class shown in boundaries and positioned between dashed lines. (**B**) Pair-wise receptive and post-receptive group analysis confirmed five post-receptive samples shown in classification boundaries represented by dashed lines.

Endometrial tissue samples from healthy volunteers (MV group) were used for classification accuracy validation. In total, tissue samples from NC ESE (n = 26), MSE (n = 26), and LSE (n = 5) phases were analysed (Table 1). The test evaluated all ESE samples as pre-receptive. Considering the MSE group, 25 samples were classified as receptive (25/26, 96.2%), from which six samples were classified as early-receptive (6/26, 23.1%) (Table 2). One sample from the MSE group (1/26, 3.8%) was classified as pre-receptive, concordant with prior histological evaluation. All LSE validation group samples were classified as post-receptive samples (Fig. 5). In conclusion, all samples with concordant histological and LH dating were classified to the expected receptivity group, while one biopsy (1/57, 1.8%) demonstrated a discrepancy with the beREADY classification. It is relevant to note that a slight WOI shift, within the normal variability of the WOI range, was detected in almost every fourth healthy woman (23.1%) in MSE (Table 2).

**Table 1:** General characteristics of study participants.

Group description	Model training and development group (MD)		Model validation group (MV)	RIF study group (RIF)
	Healthy (n = 35)	PCOS (n = 39)	Healthy (n = 31)*	RIF (n = 44)
Age (years, mean ± SD)	34.1 ± 3.7	34.5 ± 3.6	30.1 ± 3.1	35.9 ± 3.9
BMI (kg/m^2^, mean ± SD)	23.1 ± 2.5	24.7 ± 3.2	23.1 ± 4.3	23.6 ± 3.7
Proliferative (n)	10	10	–	–
Early-secretory (n)	10	10	26	–
Mid-secretory (n)	10	10	26	44
Late-secretory (n)	5	9	5	–

*BMI* body mass index, *RIF* recurrent implantation failure, *SD* standard deviation.

*Paired samples obtained from the same natural cycle in mid-secretory phase and early-secretory phase groups.

**Table 2:** Endometrial receptivity status according to the beREADY model.

Predicted receptivity group	Healthy ESE (MV, n = 26)	Healthy MSE (MV, n = 26)	Healthy LSE (MV, n = 5)	RIF MSE (RIF, n = 44)
Pre-receptive	26 (100%)	1* (3.8%)	0	3 (6.8%)
Early-receptive^1^	0	6 (23.1%)	0	8 (18.2%)
Receptive	0	19 (73.1%)	0	29 (65.9%)
Late-receptive^2^	0	0	0	0
Post-receptive	0	0	5 (100%)	4 (9.1%)

*RIF* recurrent implantation failure, *MV* model validation set, *ESE* early-secretory menstrual phase, *MSE* mid-secretory menstrual phase, *LSE* late-secretory menstrual phase.

*Out of phase biopsy.

^1^Transitionary early-receptive phase similar to the receptive group.

^2^Transitionary late-receptive phase similar to the receptive group.

### RIF group study

Finally, a group of infertile women with RIF diagnosis (n = 44) were analysed. Out of the RIF MSE samples, three patients (3/44, 6.8%) had a pre-receptive profile and displaced WOI (Table 2). No late-receptive samples were detected, but four displayed a post-receptive profile (4/44, 9.1%). Additionally, eight patients (8/44, 18.2%) were in the normal variability of the WOI range but deviated slightly from the receptive expressional profile and were classified as early-receptive. As a result, displaced WOI was detected in 15.9% of the samples in the RIF group. The proportion of the endometrial samples with the displaced WOI was higher in the RIF group than in the MV group, composed of healthy women, 15.9% and 1.8%, respectively (p = 0.012).

## Discussion

This study presents a novel tool for measuring endometrial receptivity. It is based on a highly quantitative TAC-seq assay that allows precise and cost-effective endometrial receptivity biomarker analysis. A custom computational model for classifying sequenced samples was developed to analyse data generated by the TAC-seq pipeline. As it is based on the expressional profile of a targeted set of genes, the beREADY classification model can be used in high-coverage whole-transcriptome studies. As a result, using endometrial biomarkers discovered from datasets with high predictive power for receptivity^22,24^, our prediction model allows the construction of the continuous transcriptomic states of endometrial receptivity development.

Previous transcriptomic studies of endometrium have shed light on the complex cross-talk mechanism between implanting embryos and the uterus. This insight has explained why embryo implantation in some women repeatedly fails, regardless of the use of seemingly high-quality embryos^3,14,25^. Therefore, the focus on helping RIF patients has shifted towards elucidating the maternal factors contributing to unsuccessful IVF cycles. This interest has propagated the development of transcriptomic analysis tools for determining the receptivity status of the endometrium^10,18,20,21^. Those tests use varying sets of targeted genes and different methodologies. The ERA test analyses 238 receptivity genes using microarray technology, Map^®^/ER Grade^®^ test, and ERPeakSM test analyses 40 genes and WIN-Test 11 genes with quantitative PCR. The selection of genes in the beREADY model is based on the comprehensive meta-analysis of endometrial receptivity biomarkers^22^, complemented by eleven additional genes relevant for WOI and four housekeeper genes. To our knowledge, the beREADY model is the only endometrial receptivity testing tool that applies the Unique Molecular Identifiers (UMI) technology, enabling original transcript counts estimation while avoiding the PCR-caused bias in results. Therefore, TAC-seq technology eliminates laboratory-caused PCR duplicates and counts the biomarkers at a single-molecule level^23^. This approach has already been used to determine the menstrual cycle phases from endometrial tissue^26^.

For beREADY model development, we used natural cycle endometrial samples from healthy women and women with PCOS diagnosis. Although the involvement of endometrial factors in PCOS-associated infertility has been suggested (reviewed in Piltonen, 2016)^13^, there are no large-scale and systematic studies simultaneously analysing numerous endometrial receptivity-related genes in different cycle phases in PCOS patients. In addition, the endometrial tissues of PCOS patients have demonstrated altered responses to steroids^27,28^, but the overall effect of endometrial dysfunction on pregnancy outcomes is still unclear^29^. Therefore, we tested if PCOS alters the transcriptomic profiles of our selection of receptivity biomarkers. We found no significant differences between healthy controls and PCOS patients in the expression profile of endometrial biomarkers throughout the menstrual cycle from PE to the LSE phase. Based on these findings, we concluded that the selected receptivity biomarkers are unaffected in women with PCOS, and the samples were suitable for use as a reference together with samples from healthy women in the development of the beREADY model. One of the contributing factors to why we did not see significant differences in the expressional profiles of PCOS and healthy samples is that some women in the test development group had lost their PCOS characteristics since their initial diagnosis due to ageing. Nevertheless, it must be noted that PCOS is a lifelong condition despite the effect of ageing and that a retrospective diagnosis is allowed^30^. Secondly, patients in our study were not obese. When combined, PCOS and obesity can still introduce an additional risk factor for impaired endometrial receptivity^31^. For these reasons, our conclusions regarding receptivity in PCOS patients are valid for women with PCOS patients within the normal range of BMI.

We validated the beREADY model by analysing endometrial biopsies from healthy women representing the fertile female population, displaying a high similarity between sample collection time and the molecular test result (56/57, 98.2%). This similarity can be explained by the fact that the healthy women in our validation group were all LH tested and had ultrasound confirmation of ovulation and normal hormonal values. Despite this, one MSE sample (1/26, 3.8%) with corresponding tissue morphology suggested the PE phase had shifted WOI according to the beREADY. In that case, the beREADY model classified the sample as pre-receptive, suggesting that the WOI had not yet arrived. However, displaced WOI among oocyte donors and women undergoing IVF without RIF diagnosis has been previously reported^11,32^. In the MSE group, we also classified six samples as early-receptive (6/26, 23.1%), suggesting a slight shift in WOI that remained within the normal range of receptivity. Therefore, transcriptomic profiling can offer additional accuracy for determining the individual receptivity timing of the endometrium.

The criteria for RIF diagnosis are controversial and include several factors, such as the number of failed treatment cycles, the number of transferred good-quality embryos and maternal age^33^. Due to its heterogeneous nature, the causes of RIF have remained largely unknown. When focusing solely on endometrial factors, it has been proposed by Sebastian-Leon et al. that RIF is caused by at least two distinct molecular phenomena of displaced (asynchronous) or disrupted (pathological) WOI^8^. In our study, we applied the beREADY model to detect the rate of displaced WOI in a study group of 44 RIF patients. In total, we detected shifted WOI in 15.9% of RIF cases, which is slightly less than the results described previously by Lessey et al., 25%^6^, Mahajan et al., 27.5%^34^, Hashimoto et al., 24%^9^, and Patel et al. 17.7%^25^ in women with RIF diagnosis. Compared with the MV group, the RIF study group demonstrated a significantly higher proportion of shifted WOI cases (p < 0.05), indicating that displaced WOI can cause RIF. Moreover, Eisman et al*.* described a significant decrease in ongoing pregnancy rates in the RIF cohort with asynchronous endometrium compared to control infertile patients with detected WOI displacement^35^, suggesting that there are additional factors in implantation failure beyond an adjustment in progesterone exposure. Indeed, in RIF cases, additional endometrial-related factors likely contribute to the implantation failure along the displaced or disrupted WOI, like dysbiotic vaginal or uterine microbiome^36,37^.

Evidence on the clinical effectiveness of endometrial receptivity testing on the IVF pregnancy rate is still controversial. Several studies have demonstrated a significant increase in implantation rate with pET with ERA^19,38^, Win-test^3^ and rsERT^10^ tests. It has also been proposed that receptivity testing before the first embryo transfer increases the implantation and cumulative pregnancy rates when pET is compared with conventional embryo transfers^19^. Contrary to those reports, recent randomised controlled trials (RCT) and meta-analyses question the effectiveness of endometrial testing and find its impact insignificant^39–42^. Although other similar systematic meta-analyses and single cohort studies confirm these results of no benefit of WOI testing in good prognosis patients, they also report that in the RIF subgroup, there is a significant increase in pregnancy rates following pET^38,43,44^. Another study by Haouzi et al. demonstrated that the implantation rate increased over three times, from 7 to 23%, after using the personalised WOI determination in IVF patients with RIF diagnosis^3^. Despite generally supporting endometrial testing in RIF cases, these studies also stress low certainty of evidence, thus advocating continued research on this controversial topic^38,43,44^. One reason for the disparity between studies might be that the actual rate of recurrent implantation failure due to endometrial factors may be lower than previously thought. A large-scale retrospective study involving nearly 4500 patients undergoing IVF with preimplantation genetic testing for aneuploidy (PGT-A) discovered that only around 5% of the infertile patients failed to achieve clinical pregnancy following three consecutive frozen euploid single embryo transfers^45^. Embryonic and maternal factors such as embryonal chromosomal aberrations, impaired endometrial receptivity, maternal immune dysfunction, and infertility-associated diseases, like endometriosis and male factors, can all affect the chance of embryo implantation. Therefore, due to the challenges related to the high heterogeneity of the study designs and groups, only specific subgroups of women might benefit from the endometrial receptivity testing. In summary, personalised endometrial dating likely increases the chance of implantation in patients with recurrent IVF failure. At the same time, it is considered an unnecessary ‘add-on’ for patients planning their first IVF treatment. However, the overall efficacy of the approach is still largely unclear due to the disparity between the large-scale studies and the transcriptomic tests applied.

The potential clinical utility of endometrial receptivity testing is also fully dependent upon the consistency of the gene expression profile in subsequent cycles following the endometrial biopsy. Indeed, this claim has been approved by the seminal paper of Díaz-Gimeno et al.^46^. In this study, the authors demonstrated the reproducibility of the endometrial receptivity testing, as seven women underwent ERA testing twice. The second endometrial biopsy was obtained from these women on the same day of their menstrual cycle between 29 and 40 months after the first study cycle. The intercycle variation analysis showed total consistency in the ERA test diagnoses between the same patient's first and second tests. Therefore, it is believed that the gene expression profile of the endometrial receptivity remains consistent in time, at least for the subsequent couple of months after biopsy, allowing the embryo transfer time to be adjusted according to the endometrial receptivity test recommendations. This personalised embryo transfer would allow to select the time period with the peak of endometrial receptivity for embryo transfer. However, as the initial study involved only seven patients, the question about the endometrial profile’s cycle-to-cycle consistency requires further studies.

Moreover, using the endometrial tissue biopsy for its receptivity testing would exclude the possibility of embryo transfer in the same cycle, thereby prolonging IVF treatment. Considering the possible cycle-to-cycle variation of the window of implantation timing, the endometrial receptivity testing could benefit if it could be analysed non-invasively at the cycle when embryos are planned to be transferred. Receptivity assessment from uterine fluid would circumvent the abovementioned problems, as uterine fluid aspiration can be done before embryo transfer, which is not detrimental to the implantation rate^47,48^. Recently, we provided proof for RNA biomarker-based minimally invasive endometrial receptivity testing using uterine fluid-derived extracellular vesicles and applying the beREADY endometrial testing model to successfully determine the receptivity status of the samples^49^. A similar approach would open new possibilities for non-invasive endometrial receptivity testing, which may eliminate the main shortcomings of the endometrial biopsy-based approaches in personalised embryo transfer.

Some limitations of this study should also be noted. Although the MD set consisted of samples with clearly different expressional profiles corresponding to the different menstrual cycle phases, this study suffers from a relatively small sample size with uneven distribution of samples between the receptivity classes. Furthermore, to estimate the clinical usefulness of the model, an extensive RCT must be carried out among the RIF patients, comparing the implantation, pregnancy, and live birth rates between pET and conventional embryo transfer cycles. Ideally, the RCT should be carried out with euploid embryos following PGT-A, which could rule out the genetic defects of IVF embryos as one of the possible causes of implantation failure. PGT-A has been shown to improve the cumulative IVF pregnancy outcome, avoiding the RIF diagnosis in some patients because of the transfer of the euploid embryos only^45^. Therefore, in our study, the RIF samples with possibly embryo-associated implantation failure were not excluded, likely explaining why in our findings the receptive endometrium was found in around 85% of RIF cases.

In conclusion, our results demonstrate that TAC-seq can be successfully applied for transcriptomic endometrial dating and establishing of the WOI. This assay detected distinct profiles of endometrial samples at pre-receptive, receptive, and post-receptive stages. Additionally, we found no significant difference in the expression levels of receptivity biomarkers in healthy fertile women and women diagnosed with PCOS. Based on these findings, we developed the beREADY endometrial receptivity testing tool with a custom classification model for distinguishing between the transcriptional profiles. In our study, we detected an increased rate of displaced WOI cases in the RIF study group compared to the validation group. Consequently, these findings suggest that applying personalised receptivity testing before embryo transfer could potentially reduce the chance of implantation failure in RIF patients.

## Materials and methods

All methods were carried out in accordance with relevant guidelines and regulations' or the 'Declaration of Helsinki'.

### Patient selection and sample collection

Three different study groups were used for the training and testing of the beREADY model. The general characteristics of these groups are presented in Table 1. The menstrual cycle phase was confirmed by menstrual cycle history and LH peak measurement using Clearblue^®^ digital ovulation test or BabyTime hLH urine cassette (Pharmanova). Endometrial histological evaluation of biopsies was done according to Noyes’ criteria. None of the women had been using hormonal medications for at least three months before the biopsy. All endometrial tissue biopsies were collected using a Pipelle^®^ flexible suction catheter (Laboratoire CCD, France).

#### Model training and development group

Endometrial samples in the MD group were collected from different menstrual cycle phases of the NC from healthy fertile volunteers (n = 35) and women with PCOS (n = 39) at the Oulu University Hospital (Finland), Table 1. The healthy BMI-matched controls for the PCOS group had a regular menstrual cycle without any signs of PCOS or endometriosis. The women with PCOS were identified from the hospital register based on their prior diagnosis of PCOS (Rotterdam criteria). Their current PCOS phenotype was confirmed by interview, clinical examination, and vaginal ultrasonographic examination of ovaries (Voluson 730 Expert). All women with PCOS diagnosis had ongoing or previous polycystic ovarian morphology, current or previous oligomenorrhea, and two had signs of clinical hyperandrogenism. In healthy and PCOS groups, PE, ESE, MSE, and LSE endometrial samples were collected.

#### Model validation group

The MV group consisted of 26 pairs of endometrial tissue samples from healthy volunteers at ESE and MSE phases. In addition, we included endometrial tissue samples collected from five healthy volunteers at LSE phase. Therefore, we sequenced 57 endometrial tissue samples from 31 volunteers (Table 1). The paired NC ESE and MSE samples were collected at the Nova Vita Clinic (Tallinn, Estonia), and the LSE samples were collected from Oulu University Hospital (Finland). All women had normal serum levels of progesterone, prolactin, and testosterone, negative screening results for sexually transmitted diseases, no uterine pathologies, at least one live-born child and no endometriosis or PCOS in the anamnesis. Tissue histology analysis revealed one MSE phase tissue sample belonging to PE phase, and one ESE sample had ambiguous histological morphology with minor similarities to the MSE samples. With these exceptions, the results of the histological evaluation were concordant with the time of the biopsy.

#### RIF study group

RIF group samples were obtained from women undergoing IVF at the Nova Vita Clinic in NC (n = 44, Table 1). The endometrial biopsies were collected for research purposes, and no frozen embryos were replaced in the same cycle. All RIF patients had undergone, on average, 3.8 previous unsuccessful IVF cycles with good-quality embryos, and the causes for infertility treatment were tubal (n = 15), male (n = 11), unknown (n = 8), endometriosis (n = 2), and other factors (n = 8).

### RNA extraction from endometrial tissue

According to the manufacturer’s protocol, endometrial tissue total RNA was extracted using miRNeasy or RNeasy Mini kit (Qiagen). DNase I treatment was performed on column using RNase-Free DNase Set (Qiagen). Purified RNA integrity number (RIN) and quantity were assigned with Bioanalyzer 2100 RNA Nano 6000 kit (Agilent Technologies) and Qubit RNA IQ Assay (Thermo Fisher Scientific). Samples with RIN ≥ 7 were considered eligible for further analysis.

### Biomarker selection and assay design

In total, 57 previously published biomarkers were used as endometrial receptivity biomarker genes^22^ together with four housekeeping genes *(SDHA, CYC1, TBP*, and *HMBS*)^23^ and 11 additional WOI-related genes (*CAMK2D, CAAP1, FOXN2, GGNBP2, ICA1L, LEFTY1, OGT, PPIP5K2, RIC3, TPM2*, and *YARS2*).

Highly sensitive and quantitative TAC-seq technology^23^ was applied for endometrial receptivity biomarker profiling (Fig. 1). Briefly, the TAC-seq assay was modified to analyse mRNA biomarkers based on their oligo-T primed cDNA synthesis. The robustness of the screening test was increased by designing TAC-seq specific DNA oligonucleotides as probes to be located close to the biomarker’s mRNA 3’-end, enabling higher tolerance for RNA degradation. Each biomarker molecule was detected by stringent hybridisation of two specific DNA probes close to each other on the cDNA molecule. Once the DNA oligonucleotides are hybridised, the strands are joined enzymatically. The formed complex has all the required components for further quantitative analysis, including UMI motifs (2 × 4 bp UMI barcode per complex). The application of the UMI method allows for the identification and removal of PCR duplicates in silico, enabling the quantification of transcripts of interest at a single-molecule level.

### Library preparation and sequencing

The detailed protocol of TAC-seq was published previously^23^, but critical updates were applied to develop the endometrial receptivity testing assay. For Illumina-compatible library preparation, 4 µl total-RNA (50 ng/µl) was first denatured 2 min at 80 °C and then mixed with 1 µl FIREScript^®^ RT cDNA synthesis MIX (Solis BioDyne, Estonia). One microliter of TAC-seq probe mixture was added to previously prepared cDNA. After an hour of stringent hybridisation at 60 °C, the thermostable ligase was mixed according to the protocol. Further, PCR was performed in 40 µl volume containing 1 × proofreading HOT FIREPol Blend Master Mix (Solis BioDyne) and 250 nM primers. PCR products were pooled, purified with DNA Clean & Concentrator-5 column kit (Zymo Research), and eluted with 50 µl of elution buffer (EB). Mag-Bind Total Pure NGS beads (Omega Bio-Tek) were added to 50 µl of the purified PCR product, incubated for 5 min at room temperature, and captured by a magnet for 3 min. After incubating, the supernatant was discarded, and beads were eluted in 25 µl of EB. The 180 bp uniform libraries were visualised and quantified on a TapeStation High Sensitivity D1000 ScreenTape (Agilent Technologies). High-coverage TAC-seq libraries were sequenced with Illumina NextSeq 550 high output 75 cycles kit.

### Sequencing data processing

TAC-seq sequencing data processing is described in detail^23^, and open-source software for TAC-seq data processing is available at https://github.com/cchtEE/TAC-seq-data-analysis. Firstly, the software pipeline matched sequencing reads to the target sequences in the beREADY assay. Up to five mismatches per target sequence were allowed when matching barcodes. Next, UMI thresholding was applied by merging target sequence counts with the same UMI sequence. For the sequencing assay, a UMI threshold of at least one unique UMI per target sequence was used. Finally, after obtaining the molecule count estimates, the counts were normalised considering the geometric mean of the molecule counts of the housekeeping genes. Each sample’s resulting gene expression levels were used for further downstream analysis.

### Quality control in endometrial samples

The MD group included endometrial samples collected in different menstrual cycle phases from healthy fertile volunteers (n = 35) and women with PCOS (n = 39) at the Oulu University Hospital (Finland) (Table 1). For the PCOS analyses and beREADY model development, 11 endometrial samples were excluded (2 samples from PE, 2 from ESE, 3 from MSE and 4 from LSE phase) due to uncertain sample status or quality, unclear tissue histology or insufficient sequencing data quality. As a result, endometrial samples were used from 63 women (18 samples from PE, 18 from ESE, 17 from MSE, and 10 from the LSE phase).

### Statistical analysis

Statistical analysis, model development and visualisation were done in R language (v4.1.2)^50^. Before statistical testing, shifted logarithm transform was applied to the quantified and normalised read counts. The significance of the difference in proportions of displaced WOI in MV and RIF groups was tested with a lower-tailed Fisher’s exact test. In the case of comparative analysis between two groups, an independent t-test with Bonferroni correction was applied to find significant (p < 0.05) differentially expressed genes. A two-sided analysis of variance (ANOVA) was performed when investigating the interaction of two independent variables, and the interaction score was evaluated with the F-test.

For assessing the accuracy of the computational model, fivefold cross-validation was applied. In every iteration, samples were randomly divided into five subgroups, of which four were used to train the model and one to test. At least one random sample from each group was assigned to the testing group during the stratification. The test samples were classified with the most probable receptivity class by comparing every class’s relative receptivity probability outputs. This procedure was repeated 100 times, and the average accuracy over all the classes was reported.

### beREADY computational model development

The model for detecting endometrial receptivity was based on relative cluster distances to the MD phase groups after dimensionality reduction with principal component analysis (PCA). Briefly, the MD samples were normalised with the geometric mean of housekeeper gene expression levels and scaled. Next, Horn’s parallel analysis (100 iterations; 0.05 quantile) determined the number of principal components to keep^51^. The development set eigenvectors and scaling parameters from the PCA were used to project MV or RIF samples to the previously selected principal components. After projection, the centroids for the MD receptivity phases were calculated. Subsequently, squared Mahalanobis distances of the projected samples to the reference set group centroids were calculated. Samples with p-values corresponding to the lower tail of the χ^2^ distribution smaller than 0.025 were considered outliers^52^. This procedure was repeated for the closest pair of groups. The relative probability of the test sample belonging to either of the closest groups was reported. Only distances to the closest temporally adjacent receptivity stages were compared on the second iteration, constructing a hierarchical sequence of exclusive predictive classes. The relative receptivity class (‘pre-receptive’, ‘receptive’ and ‘post-receptive’) probabilities for each studied sample were reported.

### Ethics approval

The study was approved by the Research Ethics Committee of the University of Tartu, Estonia (333/T-6) and by the Research Ethics Committee of the Hospital District of Northern Ostrobothnia, Finland (No 65/2017).

### Consent to participate

Written informed consent was obtained from all individual participants included in the study.

### Supplementary Information

Supplementary Information.

## Data Availability

The datasets generated or analyzed during the current study are available from the corresponding author upon reasonable request.
