# Supplementary material for: Targeted gene expression profiling for accurate endometrial receptivity testing
Source: Sci Rep. 2023 Aug 26;13:13959. doi: 10.1038/s41598-023-40991-z (PMC10460380; doi:10.1038/s41598-023-40991-z)
Supplement: Supplementary file 1 — Supplementary Information. [file 41598_2023_40991_MOESM1_ESM.docx]

# Supplementary Information

### Targeted gene expression profiling for accurate endometrial receptivity testing

Alvin Meltsov^Ϯ1,2^ & Merli Saare^Ϯ1,3^, Hindrek Teder^1,4^, Priit Paluoja^1,3^, Riikka K. Arffman^5^, Terhi Piltonen^5^, Piotr Laudanski^6,7,8^, Mirosław Wielgoś^9^, Luca Gianaroli^10^, Mariann Koel^11^, Maire Peters^1,3^, Andres Salumets^1,3,12^, Kaarel Krjutškov*^1,3^ and Priit Palta*^1,11,13^

##### **1** Competence Centre on Health Technologies; 50411 Tartu, Estonia **2** Department of Genetics and Cell Biology, GROW School for Oncology and Developmental Biology, Maastricht University; 6200 MD Maastricht, The Netherlands

##### **3** Department of Obstetrics and Gynecology, Institute of Clinical Medicine, University of Tartu; 50406 Tartu, Estonia

##### **4** Institute of Biomedicine and Translational Medicine, University of Tartu; 50411 Tartu, Estonia

##### **5** Department of Obstetrics and Gynecology, PEDEGO Research Unit, Medical Research Center, Oulu University Hospital, University of Oulu; FI-90014 Oulu, Finland

##### **6** Oviklinika Infertility Center; 01-377 Warsaw, Poland

##### **7** Women’s Health Research Institute, Calisia University; 62-800 Kalisz, Poland

##### **8** Department of Obstetrics, Gynaecology, Gynecology and Gynaecological Oncology, Medical University of Warsaw; 02-091 Warsaw, Poland

##### **9** Premium Medical Clinic; 04-359 Warsaw, Poland

##### **10** SISMeR, Reproductive Medicine Institute; 40138 Bologna, Italy

##### **11** Institute of Genomics, University of Tartu; 51010 Tartu, Estonia

##### **12** Division of Obstetrics and Gynecology, Department of Clinical Science, Intervention and Technology (CLINTEC), Karolinska Institutet and Karolinska University Hospital; SE-141 52 Stockholm, Sweden

##### **13** Institute for Molecular Medicine Finland (FIMM), University of Helsinki; FI-00014 Helsinki, Finland

##### Ϯ The authors consider that the first two authors should be regarded as joint First Authors

##### * The authors consider that the last two authors should be regarded as joint Last Authors

## **Supplementary Table**

**Supplementary Table I.** Gene-wise differential analysis between PCOS and healthy subjects. FDR – false discovery rate; ANOVA – analysis of variance.

| Gene | Pre-receptive P-Value | Pre-receptive FDR | Receptive P-Value | Receptive FDR | Post-receptive P-Value | Post-receptive FDR | ANOVA P-Value |
| --- | --- | --- | --- | --- | --- | --- | --- |
| *ABCC3* | 0.563 | 0.985 | 0.601 | 0.971 | 0.846 | 0.913 | 0.628 |
| *ACADSB* | 0.324 | 0.985 | 0.245 | 0.882 | 0.041 | 0.630 | 0.882 |
| *ANXA2* | 0.855 | 0.985 | 0.661 | 0.971 | 0.774 | 0.877 | 0.988 |
| *ANXA4* | 0.978 | 0.985 | 0.235 | 0.882 | 0.887 | 0.928 | 0.794 |
| *AOX1* | 0.524 | 0.985 | 0.641 | 0.971 | 0.487 | 0.715 | 0.361 |
| *APOD* | 0.629 | 0.985 | 0.149 | 0.882 | 0.451 | 0.696 | 0.364 |
| *AQP3* | 0.947 | 0.985 | 0.819 | 0.971 | 0.393 | 0.696 | 0.990 |
| *ARG2* | 0.524 | 0.985 | 0.313 | 0.882 | 0.228 | 0.630 | 0.613 |
| *ARID5B* | 0.514 | 0.985 | 0.324 | 0.882 | 0.433 | 0.696 | 0.610 |
| *BCL6* | 0.475 | 0.985 | 0.432 | 0.956 | 0.934 | 0.948 | 0.650 |
| *C10orf10* | 0.099 | 0.985 | 0.483 | 0.965 | 0.170 | 0.630 | 0.706 |
| *C1R* | 0.251 | 0.985 | 0.832 | 0.971 | 0.377 | 0.696 | 0.950 |
| *C4BPA* | 0.908 | 0.985 | 0.786 | 0.971 | 0.238 | 0.630 | 0.998 |
| *CAAP1* | 0.772 | 0.985 | 0.946 | 0.971 | 0.361 | 0.696 | 0.752 |
| *CAMK2D* | 0.759 | 0.985 | 0.767 | 0.971 | 0.361 | 0.696 | 0.646 |
| *CD55* | 0.693 | 0.985 | 0.729 | 0.971 | 0.341 | 0.696 | 0.898 |
| *CEBPD* | 0.973 | 0.985 | 0.290 | 0.882 | 0.105 | 0.630 | 0.867 |
| *CFD* | 0.517 | 0.985 | 0.926 | 0.971 | 0.125 | 0.630 | 0.782 |
| *CLDN4* | 0.733 | 0.985 | 0.189 | 0.882 | 0.603 | 0.804 | 0.733 |
| *COMP* | 0.798 | 0.985 | 0.306 | 0.882 | 0.281 | 0.681 | 0.544 |
| *CP* | 0.270 | 0.985 | 0.904 | 0.971 | 0.200 | 0.630 | 0.692 |
| *CRABP2* | 0.961 | 0.985 | 0.536 | 0.971 | 0.635 | 0.814 | 0.849 |
| *DDX52* | 0.470 | 0.985 | 0.565 | 0.971 | 0.565 | 0.784 | 0.989 |
| *DEFB1* | 0.262 | 0.985 | 0.682 | 0.971 | 0.300 | 0.685 | 0.901 |
| *DKK1* | 0.208 | 0.985 | 0.058 | 0.850 | 0.347 | 0.696 | 0.594 |
| *DPP4* | 0.512 | 0.985 | 0.926 | 0.971 | 0.183 | 0.630 | 0.458 |
| *DYNLT3* | 0.873 | 0.985 | 0.898 | 0.971 | 0.227 | 0.630 | 0.848 |
| *EDN3* | 0.487 | 0.985 | 0.321 | 0.882 | 0.831 | 0.912 | 0.921 |
| *EDNRB* | 0.983 | 0.985 | 0.296 | 0.882 | 0.471 | 0.711 | 0.558 |
| *EFNA1* | 0.703 | 0.985 | 0.928 | 0.971 | 0.166 | 0.630 | 0.978 |
| *ENPEP* | 0.395 | 0.985 | 0.847 | 0.971 | 0.155 | 0.630 | 0.265 |
| *FOXN2* | 0.384 | 0.985 | 0.938 | 0.971 | 0.753 | 0.877 | 0.401 |
| *G0S2* | 0.482 | 0.985 | 0.423 | 0.956 | 0.727 | 0.877 | 0.642 |
| *GADD45A* | 0.738 | 0.985 | 0.357 | 0.899 | 0.175 | 0.630 | 0.700 |
| *GBP2* | 0.908 | 0.985 | 0.902 | 0.971 | 0.197 | 0.630 | 0.920 |
| *GGNBP2* | 0.776 | 0.985 | 0.118 | 0.882 | 0.210 | 0.630 | 0.674 |
| *GNLY* | 0.266 | 0.985 | 0.652 | 0.971 | 0.877 | 0.928 | 0.783 |
| *GPX3* | 0.836 | 0.985 | 0.660 | 0.971 | 0.770 | 0.877 | 0.675 |
| *HABP2* | 0.101 | 0.985 | 0.920 | 0.971 | 0.087 | 0.630 | 0.579 |
| *ICA1L* | 0.400 | 0.985 | 0.095 | 0.850 | 0.214 | 0.630 | 0.072 |
| *ID4* | 0.815 | 0.985 | 0.477 | 0.965 | 0.347 | 0.696 | 0.829 |
| *IDO1* | 0.226 | 0.985 | 0.818 | 0.971 | 0.442 | 0.696 | 0.864 |
| *IGFBP1* | 0.301 | 0.985 | 0.100 | 0.850 | 0.179 | 0.630 | 0.819 |
| *IL15* | 0.170 | 0.985 | 0.283 | 0.882 | 0.496 | 0.715 | 0.771 |
| *LAMB3* | 0.165 | 0.985 | 0.815 | 0.971 | 0.664 | 0.836 | 0.866 |
| *LEFTY1* | 0.316 | 0.985 | 0.046 | 0.850 | 0.996 | 0.996 | 0.278 |
| *MAOA* | 0.772 | 0.985 | 0.528 | 0.971 | 0.420 | 0.696 | 0.930 |
| *MAP3K5* | 0.677 | 0.985 | 0.461 | 0.965 | 0.162 | 0.630 | 0.773 |
| *MMP7* | 0.985 | 0.985 | 0.090 | 0.850 | 0.165 | 0.630 | 0.625 |
| *MT1G* | 0.624 | 0.985 | 0.895 | 0.971 | 0.302 | 0.685 | 0.816 |
| *MT1H* | 0.307 | 0.985 | 0.299 | 0.882 | 0.385 | 0.696 | 0.886 |
| *NDRG1* | 0.972 | 0.985 | 0.011 | 0.761 | 0.827 | 0.912 | 0.103 |
| *NNMT* | 0.366 | 0.985 | 0.957 | 0.971 | 0.197 | 0.630 | 0.995 |
| *OGT* | 0.654 | 0.985 | 0.064 | 0.850 | 0.200 | 0.630 | 0.983 |
| *OLFM1* | 0.461 | 0.985 | 0.309 | 0.882 | 0.706 | 0.873 | 0.987 |
| *PAEP* | 0.769 | 0.985 | 0.848 | 0.971 | 0.755 | 0.877 | 0.689 |
| *PPIP5K2* | 0.700 | 0.985 | 0.256 | 0.882 | 0.156 | 0.630 | 0.675 |
| *PRUNE2* | 0.599 | 0.985 | 0.370 | 0.899 | 0.438 | 0.696 | 0.921 |
| *RIC3* | 0.374 | 0.985 | 0.750 | 0.971 | 0.627 | 0.814 | 0.295 |
| *S100P* | 0.459 | 0.985 | 0.912 | 0.971 | 0.902 | 0.930 | 0.896 |
| *SERPING1* | 0.864 | 0.985 | 0.994 | 0.994 | 0.441 | 0.696 | 0.890 |
| *SFRP4* | 0.343 | 0.985 | 0.436 | 0.956 | 0.585 | 0.795 | 0.897 |
| *SLC1A1* | 0.856 | 0.985 | 0.763 | 0.971 | 0.280 | 0.681 | 0.481 |
| *SPP1* | 0.629 | 0.985 | 0.059 | 0.850 | 0.125 | 0.630 | 0.805 |
| *TCN1* | 0.221 | 0.985 | 0.370 | 0.899 | 0.504 | 0.715 | 0.344 |
| *TPM2* | 0.505 | 0.985 | 0.549 | 0.971 | 0.241 | 0.630 | 0.499 |
| *TSPAN8* | 0.907 | 0.985 | 0.292 | 0.882 | 0.051 | 0.630 | 0.529 |
| *YARS2* | 0.517 | 0.985 | 0.200 | 0.882 | 0.040 | 0.630 | 0.305 |

## Supplementary Figures


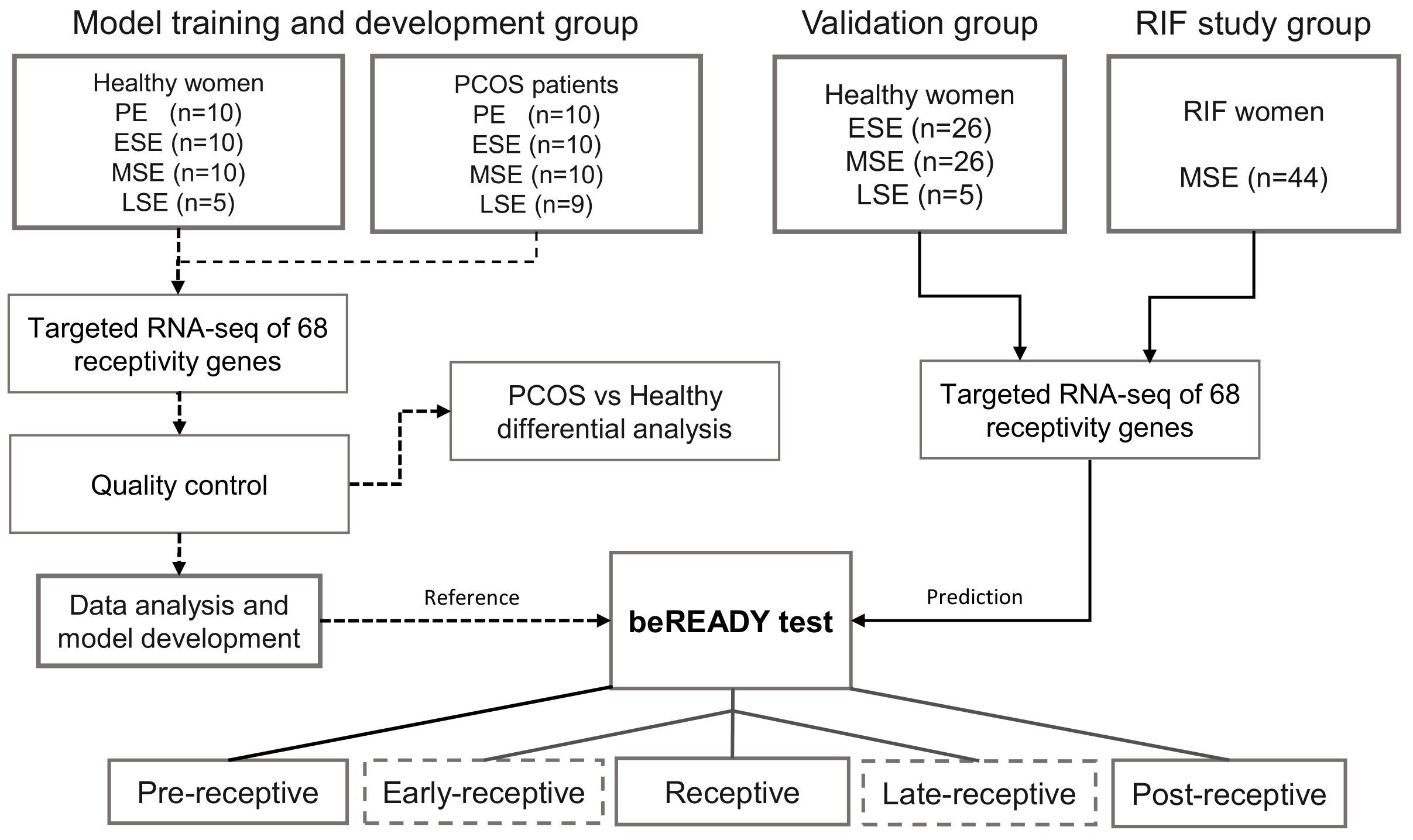


**Supplementary Figure 1. Overview of the study design.**RIF – recurrent implantation failure, PCOS – polycystic ovary syndrome, ESE – early-secretory phase, MSE – mid-secretory phase, LSE – late-secretory phase.


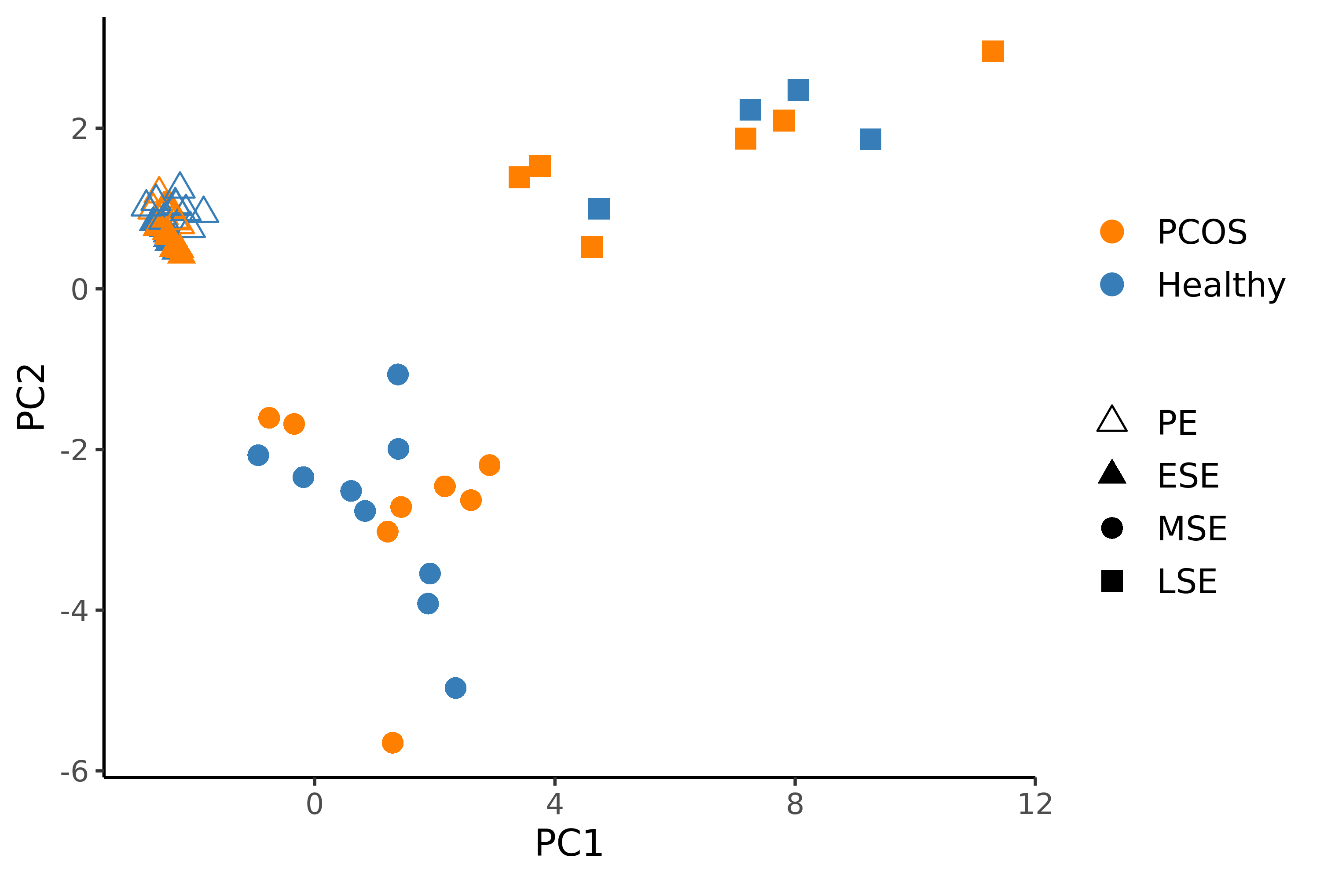


**Supplementary Figure 2. PCA plot of the model development (MD) set.**
The UMI-corrected counts were normalised with the geometric mean of housekeepers and scaled. PCOS – polycystic ovarian syndrome, PE – proliferative phase, ESE – early-secretory phase, MSE – mid-secretory phase, LSE – late-secretory phase.


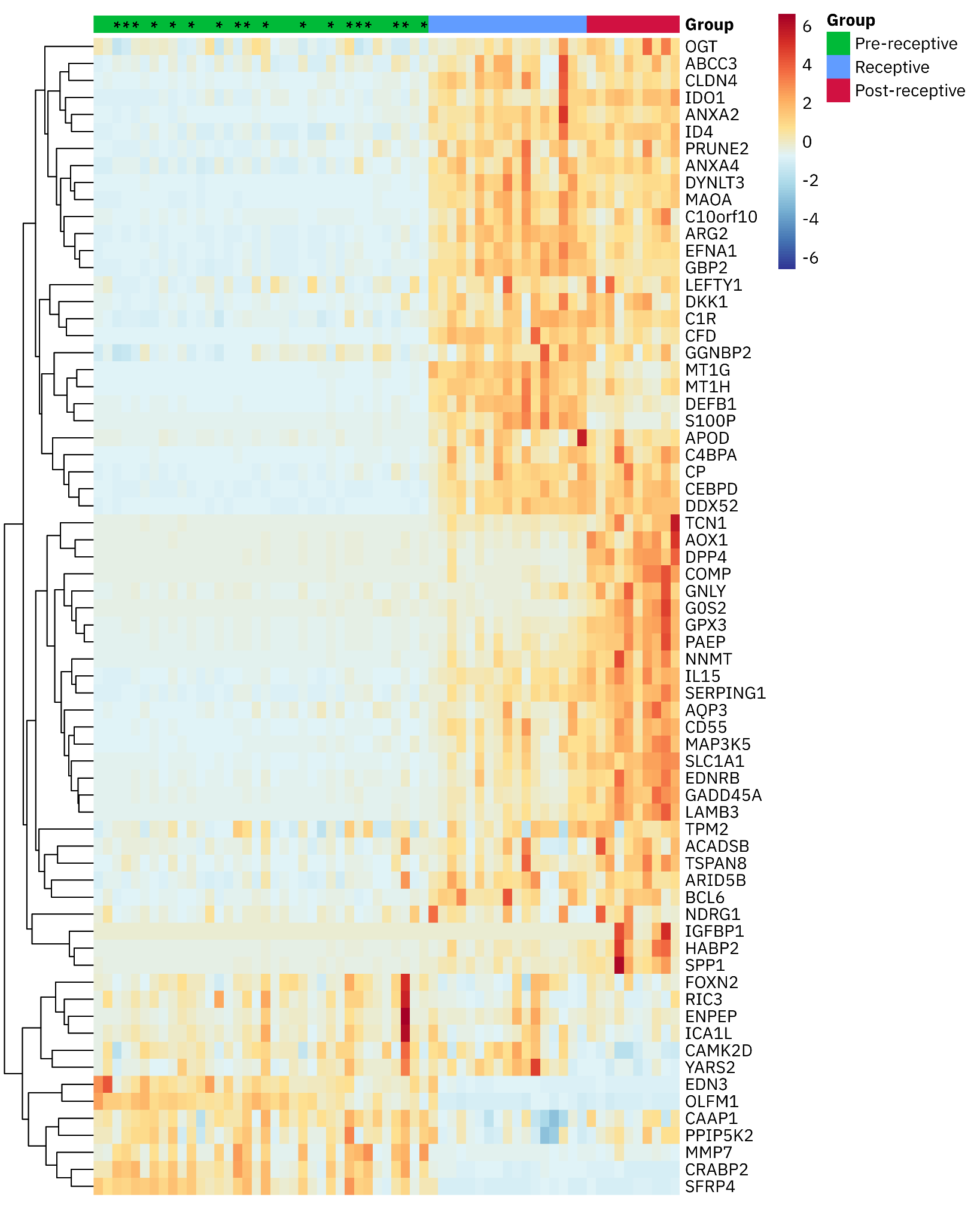


**Supplementary Figure 3. Heatmap of the scaled expression levels of normalised model training and development group samples.**Samples are ordered by the output score of the model. The genes are clustered hierarchically. Biomarker read counts are normalised with the harmonic mean of the housekeeper gene counts and gene-wise scaling was applied. Proliferative phase samples are marked with an asterisk (*).
